# Retraction: “Prevalence and associated factors of a positive *Plasmodium falciparum* antigen test among pregnant women at the Bamenda Regional Hospital, Cameroon: a cross-sectional analytical study (Pan African Medical Journal. 2024;47:99. doi: 10.11604/pamj.2024.47.99.40899)”

**DOI:** 10.11604/pamj.2024.48.168.44101

**Published:** 2024-08-09

**Authors:** The Pan African Medical Journal

**Affiliations:** 1 The Pan African Medical Journal, Yaoundé, Cameroon

**Keywords:** Retraction, double publication, research, PAMJ

## Abstract

This is to retract the published article “Prevalence and associated factors of a positive Plasmodium falciparum antigen test among pregnant women at the Bamenda Regional Hospital, Cameroon: a cross-sectional analytical study (Pan African Medical Journal. 2024;47: 99. doi: 10.11604/pamj.2024.47.99.40899)” by Dobgima Walters Pisoh, Achuo Ascensius Ambe Mforteh, William Ako Takang, Joseph Bakowe, Theodore Tameh, Merlin Boten, Audrey-Fidelia Eyere Mbi-Kobenge, Sama Julius Dohbit.

## Retraction

We, the editors of the Pan African Medical Journal would like to notify the PAMJ readers of the retraction of the article “Prevalence and associated factors of a positive *Plasmodium falciparum* antigen test among pregnant women at the Bamenda Regional Hospital, Cameroon: a cross-sectional analytical study (Pan African Medical Journal. 2024;47: 99. doi: 10.11604/ pamj.2024.47.99.40899)” by Dobgima Walters Pisoh, Achuo Ascensius Ambe Mforteh, William Ako Takang, Joseph Bakowe, Theodore Tameh, Merlin Boten, Audrey-Fidelia Eyere Mbi-Kobenge, Sama Julius Dohbit, published on Mar 1, 2024 [1].

The authors acknowledged the double publication and approved the retraction. We therefore retract this article from the literature following guidelines and best editorial practices from the Committee on Publication Ethics. We apologize to our scientific community for this unfortunate situation.

The retracted article will remain online to maintain the scholarly record, but it will be marked as “Retracted.”
